# Pulmonary Thromboembolism in a Child with Sickle Cell Hemoglobin D Disease in the Setting of Acute Chest Syndrome

**DOI:** 10.1155/2013/875683

**Published:** 2013-09-18

**Authors:** Hazel Villanueva, Sandeepkumar Kuril, Jennifer Krajewski, Aziza Sedrak

**Affiliations:** ^1^Pediatrics, The Brooklyn Hospital Center, Brooklyn, NY 11201, USA; ^2^Pediatric Hematology-Oncology, The Brooklyn Hospital Center, Brooklyn, NY 11201, USA; ^3^Hackensack University Medical Center, Hackensack, NJ 07601, USA

## Abstract

*Introduction*. Sickle cell hemoglobin D disease (HbSD) is a rare variant of sickle cell disease (SCD). Incidence of pulmonary thromboembolism (PE) and deep venous thrombosis (DVT) in children with HbSD is unknown. PE and DVT are known complications of SCD in adults but have not been reported in the literature in children with HbSD. *Case Report*. We are reporting a case of a 12-year-old boy with HbSD with acute chest syndrome (ACS) complicated by complete thrombosis of the branch of the right pulmonary artery and multiple small pulmonary artery emboli seen on computed tomography (CT) pulmonary angiogram and thrombosis of the right brachial vein seen on Doppler ultrasound. Our patient responded to treatment with anticoagulant therapy. *Conclusion*. There are no cases reported in children with HbSD disease presenting as ACS with pulmonary thromboembolism. We suggest that PE should be suspected in patients presenting with ACS who do not show improvement with standard management. CT pulmonary angiogram should be utilized for early diagnosis and appropriate management as there is no current protocol for management of PE/DVT in pediatric patients with SCD.

## 1. Introduction

 Acute chest syndrome (ACS) is one of the leading cause of morbidity and mortality in sickle cell disease (SCD). Pulmonary thromboembolism (PE) can occur as a complication of ACS or may present itself with symptoms similar to acute chest syndrome (ACS). Deep venous thrombosis (DVT) occurring in SCD due to its hypercoagulable state also predisposes to PE [1]. This report presents a case of sickle cell hemoglobin D disease (HbSD), a rare variant of SCD, with ACS complicated by PE and DVT who was treated with anticoagulants. To the best of our knowledge and extensive review of the literature, there are no cases reported in children with HbSD disease presenting as ACS with pulmonary thromboembolism.

## 2. Case Report

12-year-old male with HbSD variant, presented with complaints of fever, chest pain, and productive cough. Past medical history was significant for multiple admissions for vaso-occlusive crisis and blood transfusions.

**Figure 1:**
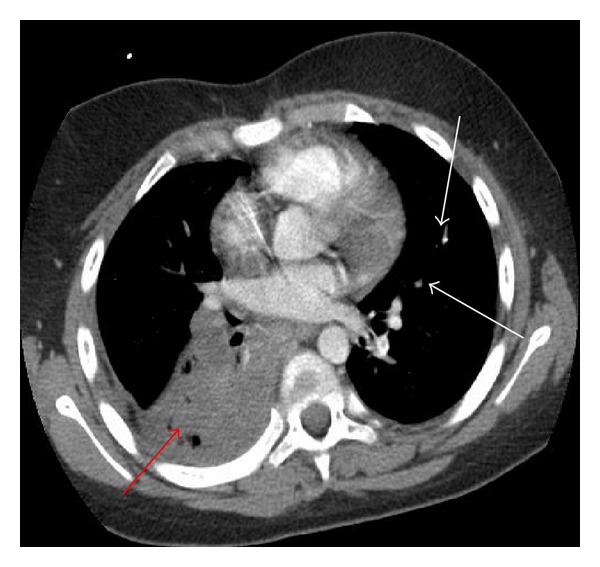
CT Chest with pulmonary angiogram. There is complete occlusion of the descending branch of the right pulmonary arteries to the right lower lobe (Red arrow). Multiple small pulmonary artery emboli were also seen in the left lobe (White arrows).

On admission, the patient was febrile (102 F), tachypneic (26 breaths/minute), with an oxygen saturation of 97% on room air (RA). His pulmonary exam was significant for bronchial breath sounds on the right lower lung fields. Chest X-ray (CXR) showed a right lower lobe (RLL) density. Patient's complete hemogram revealed Hb 9.9 g/dL, WBC 28.5 × 10^3^/*μ*L, and platelets 483 × 10^3^/*μ*L. He was given IV antibiotics, maintenance intravenous (IV) fluids, and patient controlled analgesia (PCA) with morphine. On day 2 of hospitalization, he was given supplemental oxygen for desaturations. Repeated CXR showed a new right basal process. Repeated CBC showed WBC 11.8 × 10^3^/*μ*L and Hb 7.5 g/dL. Two units of packed RBCs were transfused and his Hb increased to 10.5 g/dL. On day 7, the patient complained of increased chest pain with worsening productive cough and blood tinged sputum. His tachypnea worsened and oxygen saturation dropped to 80% on RA. Repeat CXR showed increased congestive changes without evidence of acute infiltrates. Chest CT scan with contrast was obtained which showed complete thrombosis of the branch of the pulmonary artery to the right lower lobe with subsequent lung infarction and multiple small pulmonary artery emboli in the left lobe with small areas of infarction involving left lower lobe, portions of the lingula and middle lobe; findings were confirmed by CT pulmonary angiogram (Figure 1). Thrombophilia work up for protein C & S; antithrombin III and Factor V Leiden were negative. Echocardiogram showed very small pericardial effusion with normal cardiac function. Exchange transfusion was done, and heparin drip was started after obtaining baseline coagulation studies. Effect of anticoagulation was monitored with a PTT every 6 hours. On day 9, patient developed swelling of the right upper arm. Doppler ultrasound of the right upper extremity showed DVT in the right brachial vein and superficial venous thrombosis in the right basilic vein. The dose of heparin was then increased. When the therapeutic level was reached, on day 11, heparin was tapered, and subcutaneous enoxaparin was initiated at 1.5 mg/kg. Factor Xa levels were sent to monitor anticoagulation with enoxaparin. The patient's blood and central line cultures were negative. On day 15, the patient symptomatically improved. He was weaned off oxygen, and repeated CXR showed improvement in the left lung consolidation. Repeated CBC showed Hb 12.4 g/dL and WBC 15.5 × 10^3^/*μ*L. He continued to improve, so the morphine PCA was changed to oral oxycodone, and he was discharged home on day 20.

## 3. Discussion

Sickle cell hemoglobin D is a rare variant of SCD. They have severe hemolytic anemia with a peripheral blood smear comparable to that seen in SCD and also have severe vaso-occlusive complications [2, 3]. 

In patients with SCD, the acute chest syndrome is commonly precipitated by infection, especially community-acquired pneumonia. Treatment with transfusions and bronchodilators improves oxygenation, and with aggressive treatment, most patients who have respiratory failure recover. This case presents a unique scenario whereby the patient with HbSD was diagnosed with acute chest syndrome, but despite appropriate treatment, his clinical condition deteriorated, and he was subsequently found to have PE and DVT. 

Identifiable risk factors for PE include central venous catheter, malignancy, surgery, infection, trauma, and congenital hypercoagulable disorders [4]. DVT also predisposes to PE, with DVT in the upper body being more commonly associated with children [5]. Children with SCD are prothrombotic and are at an increased risk of thromboembolism [4]. This is due partly to an acquired or inherited deficiency of natural anticoagulants (protein C, protein S, and antithrombin III) and increased coagulation activation (increased platelet activity, increased thrombin generation, and elevation of *d*-dimers) [6].

Stein et al. found that asymptomatic patients with Hb AS also experienced an increased risk of thromboembolic events [7], thereby suggesting that the increased risk associated with SCD is attributable, at least in part, to the effect of sickling erythrocytes on coagulation rather than to secondary health conditions [6].

The symptoms of thromboembolism in SCD patients with acute chest syndrome are difficult to differentiate from the similar symptoms of painful thoracic crises and infectious pulmonary episodes [8]. Our patient was diagnosed to have acute chest syndrome and in spite of receiving appropriate treatment had worsening of infiltrates on subsequent chest radiograph coupled with clinical deterioration and subsequently was found to have PE and DVT. 

A study conducted by Raffini et al. demonstrated a steady increase in the rate of venous thromboembolism in children's hospitals in the United States from 2001–2007 [9], although the incidence of PE and DVT in children with HbSD is unknown. In an adult study, prevalence of apparent PE in patients with SCD was higher compared with non-SCD patients of the same age [6, 7]. 

There is no current protocol on how to manage pediatric patients with SCD who developed PE [10]. A study by Amancio et al. reported a case of hemoglobin SC complicated by fatal pulmonary thromboembolism diagnosed at autopsy [11]. In our patient, there was a high risk of critical comprise of pulmonary function; therefore, anticoagulation with heparin was started followed by maintenance anticoagulation therapy with enoxaparin. Enoxaparin was continued for 6 months post PE, and repeated imaging by chest CT and Doppler scan of the upper extremity revealed resolution of the DVT/PE. The patient in this case seemed to respond well to anticoagulation therapy. 

We suggest that PE should be suspected in patients presenting with ACS who do not show improvement with standard management. CT pulmonary angiogram should be utilized for early diagnosis and appropriate management as there is no current protocol for management of PE/DVT in pediatric patients with SCD. Studies analyzing the prevalence of PE in children with SCD and its rare variant like HbSD, and extending it further to examining the use of anticoagulants and thrombolytics in children, are needed for the development of standardized management guidelines for PE in children with SCD and its variants.
